# Effects of rehydration nutrients on H_2_S metabolism and formation of volatile sulfur compounds by the wine yeast VL3

**DOI:** 10.1186/2191-0855-1-36

**Published:** 2011-11-02

**Authors:** Gal Winter, Paul A Henschke, Vincent J Higgins, Maurizio Ugliano, Chris D Curtin

**Affiliations:** 1 School of Biomedical and Health Sciences, College of Health and Science, University of Western Sydney, NSW, Australia; 2 The Australian Wine Research Institute, P.O. Box 197, Glen Osmond, Adelaide, SA 5064, Australia; 3 Ramaciotti Centre for Gene Function Analysis, School of Biotechnology and Biomolecular Sciences, University of New South Wales, NSW, Australia; 4 Nomacorc SA, 2260 route du Grès, 84100 Orange, France

**Keywords:** Rehydration, yeast, nutrients, H2S, hydrogen-sulfide, GSH, glutathione

## Abstract

In winemaking, nutrient supplementation is a common practice for optimising fermentation and producing quality wine. Nutritionally suboptimal grape juices are often enriched with nutrients in order to manipulate the production of yeast aroma compounds. Nutrients are also added to active dry yeast (ADY) rehydration media to enhance subsequent fermentation performance. In this study we demonstrate that nutrient supplementation at rehydration also has a significant effect on the formation of volatile sulfur compounds during wine fermentations. The concentration of the 'fruity' aroma compounds, the polyfunctional thiols 3-mercaptohexan-1-ol (3MH) and 3-mercaptohexyl acetate (3MHA), was increased while the concentration of the 'rotten egg' aroma compound, hydrogen sulfide (H_2_S), was decreased. Nutrient supplementation of the rehydration media also changed the kinetics of H_2_S production during fermentation by advancing onset of H_2_S production. Microarray analysis revealed that this was not due to expression changes within the sulfate assimilation pathway, which is known to be a major contributor to H_2_S production. To gain insight into possible mechanisms responsible for this effect, a component of the rehydration nutrient mix, the tri-peptide glutathione (GSH) was added at rehydration and studied for its subsequent effects on H_2_S formation. GSH was found to be taken up during rehydration and to act as a source for H_2_S during the following fermentation. These findings represent a potential approach for managing sulfur aroma production through the use of rehydration nutrients.

## Introduction

In many viticultural regions the natural nutrient composition of grape juice is considered suboptimal and may lead to a variety of fermentation problems including slow or stuck fermentations and formation of undesirable off-flavours (Blateyron and Sablayrolles 2001; Henschke and Jiranek 1993; Mendes-Ferreira et al. 2009; Sablayrolles et al. 1996; Schmidt et al. 2011; Torrea et al. 2011; Ugliano et al. 2010). To alleviate these deficiencies, various yeast nutrient preparations are often added to the juice prior to or during alcoholic fermentation, to contribute to the production of a quality wine. Among the nutrient supplements allowed by wine regulatory authorities in many countries are vitamins, inorganic nitrogen, usually in the form of diammonium phosphate (DAP) and organic nutrient preparations. The latter are typically prepared from inactive or autolysed yeast and are therefore usually composed of lipids, micro- and macro-elements, amino nitrogen, mannoproteins and insoluble material (for example see Pozo-Bayón (2009). Effects of these nutrients on the formation of key aroma groups in wine have been studied widely. The concentration of esters and higher alcohols, which impart fruity and fusel aromas respectively, were found to be influenced mostly by nitrogen availability (reviewed by Bell and Henschke (2005). Nitrogen is also considered a key modulator in the formation of volatile sulfur compounds, including H_2_S, a highly potent compound which possesses an odour reminiscent of rotten egg (Rauhut 1993).

The majority of studies regarding the effect of nutrients on yeast derived aroma compounds have focused on nutrient addition to the grape juice immediately prior to or during alcoholic fermentation. The common oenological practice of using active dry yeast (ADY) for wine fermentation necessitates rehydration, since water availability in ADY is too low for yeast to maintain metabolic activity during storage (Rapoport et al. 1997). This step represents a further opportunity for nutrient supplementation. Previous studies have demonstrated the efficacy of nutrient supplementation at this point in time on yeast viability and vitality. Supplementation of organic nutrient in the form of inactive dry yeast (IDY) was found to increase fermentation rate, supposedly due to an incorporation of solubilised sterol present in IDY (Soubeyrand et al. 2005). Additions of fermentable carbon source and magnesium salts were also shown to enhance both viability and vitality of dehydrated yeast following rehydration (Kraus et al. 1981; Rodríguez-Porrata et al. 2008).

Although rehydration nutrient supplementation is a common practice in winemaking, its effect on the formation of fermentation derived aroma compounds has not been explored. In this paper we examine the effect of a proprietary rehydration nutrient supplement on yeast gene expression during wine fermentation and how this affects its volatile chemical composition. This parallel analysis consisting of transcriptomics and metabolite profiling provided insights into which components of the rehydration nutrient mixture affect the formation of aroma compounds.

## Materials and methods

### Chemicals

Analytical reagents were purchased from Sigma-Aldrich unless otherwise specified. Rehydration nutrient mix was Dynastart (Laffort Australia, Woodville, SA, Australia). S-3-(hexan-1-ol)-L-cysteine (Cys-3MH) and S-4-(4-methylpentan-2-one)-L-cysteine (Cys-4MMP) were synthesized and characterized as previously described (Howell et al. 2004; Pardon et al. 2008).

### Yeast strain, treatments and fermentation conditions

The yeast strain used was a commercial active dried preparation of VL3 (Laffort Australia, Woodville, SA, Australia). ADY were rehydrated with water or water supplemented with rehydration nutrient mix (120 g/L). To examine the effect of nutrient mix components ADY were rehydrated with water containing GSH (500 mg/L). Rehydration media were thoroughly mixed at 37°C for 30 minutes prior to addition of 10% (w/v) ADY. ADY were incubated with agitation in the rehydration media for 20 minutes and then inoculated into the fermentation media to give a cell concentration of 1 × 10^6 ^cells/ml. Fermentations were carried out in triplicate under isothermal conditions at 22°C with agitation. Fermentations were carried out in Schott bottles (SCHOTT Australia, NSW, Australia), silled with silicone o-ring and fitted with silver nitrate detector tubes for the quantification of H_2_S formed in fermentation and a sampling port. Samples were collected through the sampling port using a sterile syringe. Fermentation volume was either 2 L (for comprehensive volatile analysis) or 1 L. Fermentation progress was monitored by measurement of residual glucose and fructose using an enzymatic kit (GF2635, Randox, Crumlin, UK).

### Fermentation media

A low nitrogen Riesling juice with a total yeast assimilable nitrogen (YAN) concentration of 120 mg/L (NH_3 _= 53 mg/L; free amino nitrogen (FAN) = 90 mg/L) was used for this study. Juice analytical parameters were as follows: pH, 2.9; titratable acidity 4.6 g/L as tartaric acid; sugars, 205 g/L. To examine the effect of rehydration nutrients on polyfunctional thiol release, juice was supplemented with 5 μg/L Cys-4MMP and 200 μg/L Cys-3MH, a concentration of precursors commonly found in Sauvignon Blanc juices (Capone et al. 2010; Luisier et al. 2008). Where specified, DAP addition to the fermentation media was 0.56 g/L to increase the juice YAN value to 250 mg N/L. The pH of the fermentation medium was readjusted to 2.9 with 1 M HCl following DAP additions. Juice was filter sterilized with a 0.2 μm membrane filter (Sartorius Australia, Oakleigh, Victoria, Australia).

### Post fermentation handling

At the end of grape juice fermentation, wines were cold settled at 4°C and free SO_2 _of the finished wine was adjusted to 45 mg/L by the addition of potassium metabisulfite. The wines were then carefully racked into glass bottles to avoid exposure to oxygen and were sealed with air tight caps fitted with a polytetrafluoroethylene liner. Bottles were fully filled to avoid any headspace oxygen.

### Grape juice analyses

Titratable acidity, FAN, and ammonia were measured as previously described (Vilanova et al. 2007). Ammonia concentration was measured using the Glutamate Dehydrogenase Enzymatic Bioanalysis UV method (Roche, Mannheim, Germany). FAN was determined by using the o-phtalaldehyde/N-acetyl-L-cysteine spectrophotometric assay procedure. Both ammonia and FAN were analyzed using a Roche Cobas FARA spectrophotometric autoanalyzer (Roche, Basel, Switzerland). Amino acid analysis was carried out based on Korös et al. (2008) using a pre-column derivitisation with o-phthalaldehyde-ethanethiol-9-fluorenylmethyl chloroformate and HPLC analysis with fluorescence detection. Reduced and oxidized glutathione were analyzed using LC-MSMS as previously described (du Toit et al. 2007).

### Volatile compounds analyses

H_2_S, methanethiol (MeSH), dimethyl sulfide (DMS), methyl thioacetate (MeSAc), and ethyl thioacetate (EtSAc) were determined by static headspace injection and cool-on-column gas chromatography coupled with sulfur chemiluminescence detection (GC-SCD), as described in Siebert et al. (2010). 3MH, 3MHA and 4-Mercapto-4-methylpentan-2-one (4MMP) were measured in SARCO Laboratories (Bordeaux, France) according to Tominaga et al. (2000) using a TRACE GC-MS (ThermoFisher Scientific, MA, USA). Detection limits for 3MH, 3MHA and 4MMP were 11 ng/L, 1 ng/L and 0.3 ng/L, respectively. Quantification limit is 35 ng/L ± 20% for 3MH, 3 ng/L ± 18% for 3MHA and 0.6 ng/L ± 14% for 4MMP. Monitoring of H_2_S development during fermentation was carried out using silver nitrate selective gas detector tubes (Komyo Kitagawa, Japan), as described by Ugliano and Henschke (2010).

### RNA Extraction and cDNA synthesis

Samples for RNA analyses were collected by filtration during fermentation after consumption of 15 g/L sugars. Cells were resuspended in RNAlater^® ^(Ambion, Inc., Austin, TX, USA) solution at 4°C for 24 hours. Cells were then centrifuged to remove the RNAlater^® ^solution and were stored at -80°C. Total RNA was isolated using TRIzol™ Reagent (Invitrogen, Carlsbad, CA) as described in Alic et al. (2004). The integrity of the RNA was analyzed using an RNA 6000 Nano LabChips on a Bioanalyzer 2100 (Agilent Technologies, Santa Clara, CA). cDNA was synthesized from 200 ng total RNA in a total volume of 20 μl with AffinityScript QPCR cDNA synthesis kit (Statagene, Agilent Technologies, Santa Clara, CA) and oligo-dT20 primers by incubation for 5 min at 42°C and 15 min at 55°C with heat inactivation for 5 min at 95°C.

### Transcription analyses

Transcription analysis was carried out at the Ramaciotti Centre for Gene Function Analysis (UNSW, Sydney, Australia). Biological duplicates were analysed using the Affymetrix GeneChip Yeast Gene 1.0 ST Array and the GeneChip^® ^3' IVT Express protocol (Affymetrix, Santa Clara, CA, USA). Data were analysed using the statistical methods available in the Partek^® ^Genomic Suite 6.5 (Partek Incorporated, St Louis, Missouri, USA). Statistical analysis for over-representation of functional groups was performed using FunSpec (Robinson et al. 2002). Available databases were addressed by using a probability cutoff of 0.01 and the Bonferroni correction for multiple testing. To validate the results, five differentially expressed genes were further examined by quantitative real-time PCR (qPCR). qPCR was carried out with Brilliant II SYBR Green reagent (Statagene, Agilent Technologies) and cDNA made from 2.5 ng total RNA in a volume of 25 μl for all subsequent reactions. Primers are detailed in table 1. Ct values were obtained from triplicate fermentations and were normalized using the 2^-ΔΔCt ^method (Wong and Medrano 2005). Values were then normalized against a geometric average of two reference genes obtained from geNorm (Vandesompele et al. 2002). Selection of the reference genes was based on the microarray results using an algorithm described in Popovici et al. (2009). Each individual PCR run was normalized with an intercalibration standard.

**Table 1:** qRT-PCR primers sequences

Gene	Primer sequence
***GPM1***	GCTCACGGTAACTCCTTG
	AGATGGCTTAGATGGCTTC

***TDH3***	GCTGCCGCTGAAGGTAAG
	CGAAGATGGAAGAGTGAGAGTC

***OPT1***	TGTCCCGATTGGTGGTATTTAC
	GTGTTGGTTAGTCATTGCTTCC

***MET10***	CACTCACGTTCCATCCACTACC
	CACTCACGTTCCATCCACTACC

***IRC7***	CCTGGATTTGGCTGCTTGG
	AGAACCTTTGTAGTCACGAACC

### Determination of glutathione

For the extraction of cellular glutathione, cells (100 mg) were washed three times with sodium-phosphate buffer (PBS, pH 7.4) and resuspended in 1 ml 8 mM HCl, 1.3% (w/v) 5-sulphosalicylic acid for 15 min at 4°C. Cells were then broken by vortexing at 4°C with 0.5 g of glass beads in four series of 1 min alternated with 1 min incubation on ice. Cell debris and proteins were pelleted in a microcentrifuge for 15 min (13000 rpm at 4°C), and supernatants were used for glutathione determination. For total GSH determination supernatant was used directly in 200 μl of total volume reaction as described in (Griffith 1980).

## Results

### Rehydration nutrient effect on wine volatile composition

**Figure 1:**
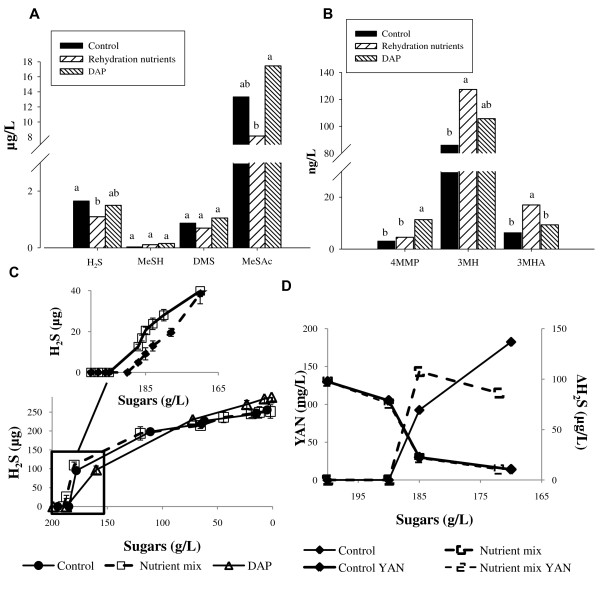
**Effects of nutrients addition on the final concentration of volatile sulfur compounds (A) and polyfunctional thiols (B)**. Nutrient treatments included supplementation of rehydration nutrients to the rehydration media (nutrient mix) or supplementation of DAP to the fermentation media (DAP) or no nutrients addition (control). Letters represent statistical significance at the 95% confidence level, as tested by Student t statistical test. C Profile of H_2_S production in the headspace during fermentation. Upper panel shows a more detailed profile of H_2_S formation in the early stage of a separate fermentation experiment. H_2_S formation was measured using gas detection tubes D H_2_S formation and YAN consumption profile during the early stages of fermentation. Fermentations were carried out in triplicate, error bars represent standard deviation.

To assess the effect of rehydration nutrients on fermentation derived aroma compounds we fermented grape juice using ADY rehydrated in either water or a commercially available rehydration nutrient mixture. Rehydration nutrient mix was prepared from inactivated yeast and contained an organic nitrogen source (mostly as amino acids) in addition to other yeast constituents including vitamins and lipids. As an additional point of reference we included inorganic nitrogen in the form of DAP added directly to the fermentation media. DAP addition to the fermentation media is a common practice among winemakers and its effects on wine aroma composition have been studied widely (Bell and Henschke 2005). Resultant wines were analysed for volatile chemical composition (Figure 1). The concentration of the polyfunctional thiols 3MH and 3MHA increased with the addition of rehydration nutrient while the concentration of hydrogen sulfide was significantly decreased. Other sulfur compounds including 4MMP were not affected by addition of nutrients to the rehydration media and we did not observe an effect on production of esters, higher alcohols and acids (p > 0.05) (Additional file 1). Rehydration nutrient supplementation also had no effect on growth rate or fermentation kinetics (data not shown). Addition of DAP stimulated growth and fermentation rates and resulted in an increased concentration of the polyfunctional thiol 4MMP (Figure 1) and acetate esters (Additional file 1), while the concentration of higher alcohols was decreased (Additional file 1). Further characterisation of the effect of rehydration nutrients on the formation of volatile sulfur compounds was obtained by monitoring H_2_S production throughout fermentation. Addition of rehydration nutrients resulted in an earlier onset and increased initial production of H_2_S while DAP addition delayed the liberation of H_2_S (Figure 1c). To test whether the rehydration nutrient effect could be attributed to YAN availability we compared the fermentation YAN concentration following ADY rehydration with either water or nutrient supplementation. As shown in Figure 1d, both treatments exhibited the same YAN consumption rate. Therefore, the increased initial production of H_2_S was not correlated with available nitrogen concentration during fermentation.

### Rehydration nutrient effect on gene expression profile

**Figure 2:**
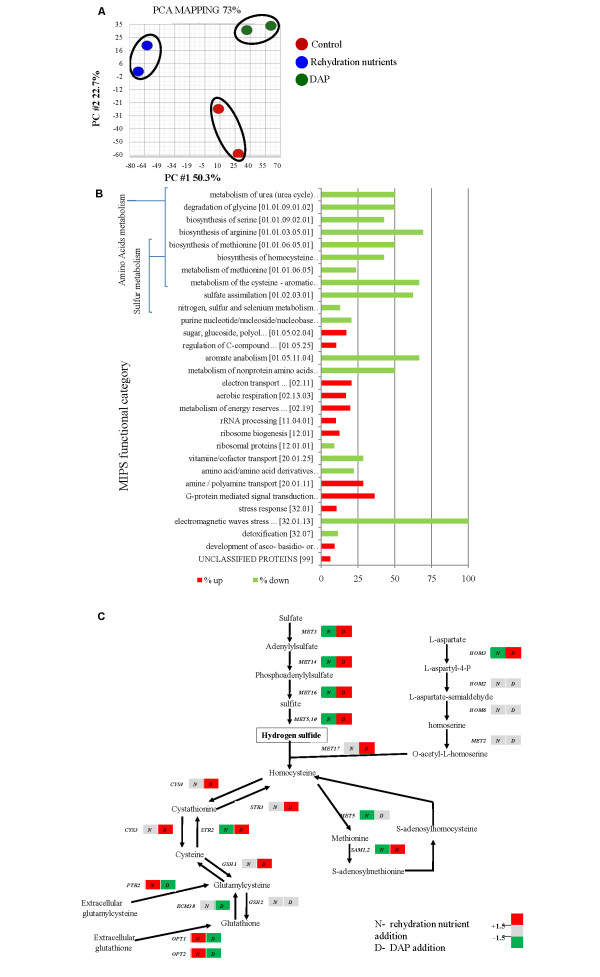
**Effect of rehydration nutrient and nitrogen supplementation upon the transcriptome.** (A) Biplot of a principal component analysis performed on the interaction between the factor gene and treatment. All 10,928 probe sets from the datasets were used in the analysis. (B) Classification of the genes affected by the rehydration nutrient addition to MIPS functional categories. Bars represent percentage of affected genes out of total genes in category. (C) Schematic representation of the sulfur metabolism pathway and its regulation by the two nutrient treatments (N- rehydration nutrient addition, D- DAP addition) in comparison to the control treatment.

To gain insight into how rehydration nutrients affect H_2_S formation we performed a global transcription analysis for each of the treatments. RNA was extracted from yeast samples taken after consumption of approximately 15 g/L of sugar from the grape juice. This sampling time corresponded with the initial increase in H_2_S due to addition of rehydration nutrient (Figure 1c). Overall analysis of the data revealed two principal components explaining 73% of the variation in gene expression (Figure 2a). This distribution is indicative that DAP and the rehydration nutrient mix had distinct effects upon the transcriptome. Classification of the genes to MIPS functional categories (Robinson et al. 2002) revealed that both treatments affected the same groups of genes, therefore the variation explained by the PC analysis was due to differential effects upon the same metabolic pathways (Figure 2b).

Addition of the rehydration nutrient mix downregulated the expression of genes involved in the biosynthesis of different amino acids and vitamin/cofactor transport (Figure 2b), consistent with its composition in these nutrients. Interestingly, amongst the downregulated genes were those involved in H_2_S production through the biosynthesis of the sulfur-containing amino acids and the sulfate assimilation pathway (Figure 2c). Addition of DAP, on the other hand, upregulated approximately 67% of the genes involved in sulfate assimilation and the synthesis of the sulfur-containing amino acids (Figure 2c). This appears to conflict with our phenotypic observations at the sampling point where the addition of rehydration nutrients induced the formation of H_2_S while the addition of DAP delayed it (Figure 1c). Nonetheless, these results support our previous hypothesis of distinct effects for each of the treatments and further suggest the presence of an additional nutrient factor regulating the formation of H_2_S.

**Figure 3:**
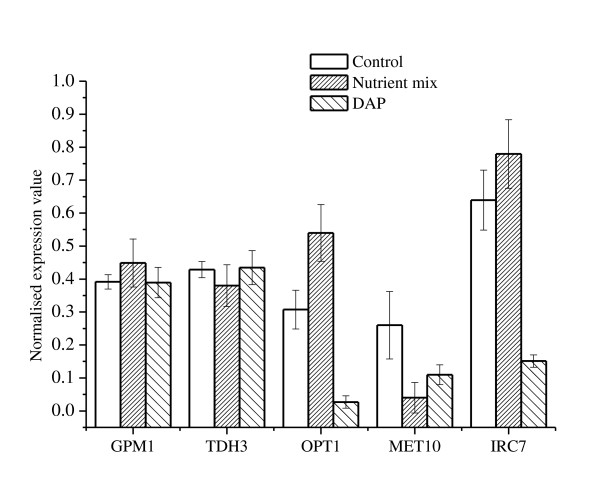
**qRT-PCR analysis of *GPM1*, *TDH3*, *OPT1*, *MET10 *and *IRC7 *mRNA level**. Expression values were calculated using the 2^-ΔΔct ^method and normalised to the reference genes *GPM1 *and *TDH3*. Fermentations were carried out in triplicate, error bars represent standard deviation.

Confirmation of the microarray results was obtained by an independent transcription analysis using qRT-PCR for samples taken at the same point in time used for the microarray analysis. *GPM1 *and *TDH3 *were selected as reference genes based on data obtained from the microarray analyses where both genes were shown to have high expression values and minimal variation between the different treatments. Genes related to sulfur metabolism that exhibited different trends of expressions between the treatments were chosen for validation (genes and primers are listed in Table 1). Consistent with transcriptomic data, *GPM1 *and *TDH3 *transcript levels were similar for all treatments. *OPT1 *was upregulated by 1.75 fold with the addition of rehydration nutrient mix and downregulated by 11 fold following DAP addition. *MET10 *was downregulated under all nutrient treatments and *IRC7 *was downregulated by 4.2 fold with the addition of DAP, consistent with its regulation by nitrogen catabolite repression (Scherens et al. 2006; Thibon et al. 2008) (Figure 3).

### Nutrient regulation of H_2_S formation

**Figure 4:**
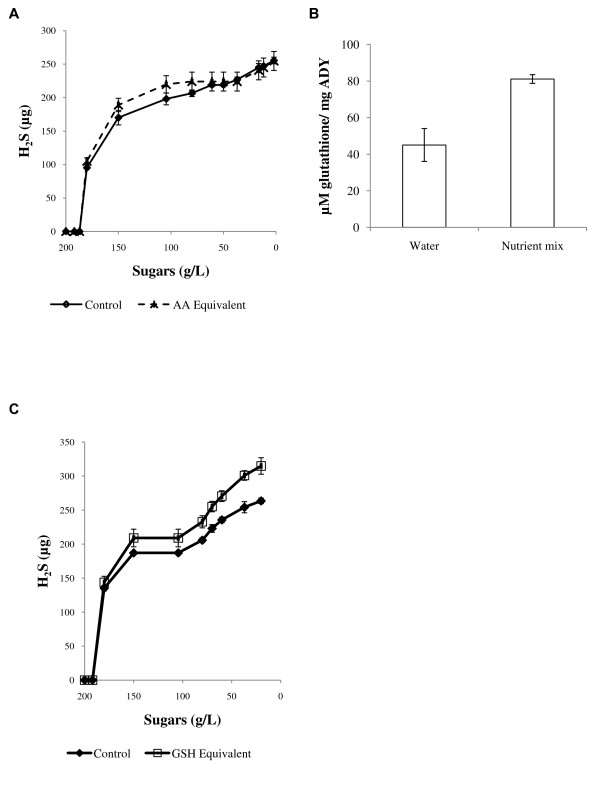
**Amino acid and GSH supplementation during rehydration. A**. Profile of H_2_S production in the headspace during fermentation following rehydration with a laboratory-made amino acids solution equivalent to the amino acid component of the rehydration nutrient mix. **B**. GSH cellular content of ADY following rehydration with water or rehydration nutrient mix. Experiments were conducted in triplicates; results are presented as percentage of the control treatment. **C**. Profile of H_2_S production in the headspace during fermentation following rehydration with 500 mg/L GSH. All fermentations were conducted in triplicates. H_2_S formation was measured using gas detection tubes Error bars represent standard deviation.

Aside from being affected by the general YAN concentration of the media, H_2_S formation is regulated by the presence of specific amino acids (Duan et al. 2004; Jiranek et al. 1995; Li et al. 2009). We therefore evaluated whether the source for the initial increase in H_2_S production, which was observed following rehydration with nutrients, was the amino acid component of the mixture (detailed in Table 2). Rehydration in a solution containing an amino acid composition equivalent to the nutrient mix did not significantly affect the kinetics of H_2_S formation (Figure 4a). This result suggests that amino acids were not responsible for altered H_2_S formation kinetics following rehydration nutrient supplementation.

**Table 2:** Rehydration nutrient mix amino acid composition

	Concentration at the rehydration media (mg/L)	Concentration at the fermentation media (mg/L)
**Alanine**	482.7	1.20675

**Arginine**	154.3	0.38575

**Asparagine**	122.6	0.3065

**Aspartic Acid**	113.7	0.28425

**Citrulline + Serine**	101.8	0.2545

**Cystine**	Not Detected	

**Gamma Amino Butyric Acid**	149.7	0.37425

**Glutamic Acid**	1218.6	3.0465

**Glutamine**	1593.6	3.984

**Glycine**	143.0	0.3575

**Histidine**	Not Detected	

**Hydroxyproline**	3.6	0.009

**Isoleucine**	92.2	0.2305

**Leucine**	135.2	0.338

**Lysine**	94.6	0.2365

**Methionine**	23.9	0.05975

**Ornithine**	198.8	0.497

**Phenylalanine**	88.4	0.221

**Proline**	209.3	0.52325

**Threonine**	73.2	0.183

**Tryptophan**	23.5	0.05875

**Tyrosine**	53.1	0.13275

**Valine**	186.3	0.46575

*'Glutathione equivalent (GSH+GSSG)	516	1.29

Another nutrient that is a potential source for H_2_S formation is the tripeptide glutathione (GSH) (Hallinan et al. 1999; Rauhut 2008; Sohn and Kuriyama 2001; Vos and Gray 1979), which can also serve as a source of organic nitrogen (Mehdi and Penninckx 1997). Analysis of the rehydration nutrient mixture revealed it contained a concentration of 500 mg/L glutathione equivalent (GSH + GSSG). Furthermore, GSH cellular content of ADY following rehydration with the nutrient mixture was ca. 1.8 fold higher than those rehydrated with water (Figure 4b). Addition of GSH as a sole nutrient during rehydration led to a significant change in H_2_S formation kinetics and a higher cumulative concentration of H_2_S produced during fermentation (Figure 4c). This confirms that GSH, taken up during rehydration, acts as a modulator of H_2_S production during fermentation.

## Discussion

Supplementation of ADY rehydration mixture with nutrients has become a common practice amongst winemakers because it generally improves yeast fermentation performance in suboptimal juices. In this study we compared the volatile composition of wines prepared from a low YAN juice by fermentation with ADY rehydrated with either a commercially available rehydration nutrient mixture or water. We found that the presence of rehydration nutrients affected the concentration of volatile sulfur compounds produced during fermentation (Figure 1) and the regulation of genes involved in sulfur metabolism (Figure 3). Importantly, the sheer nutrient contribution of the rehydration mix that was added with the ADY at inoculation did not have an effect on the wine volatile composition (data not shown).

Sulfur compounds exert a strong influence on wine aroma, due to their low detection threshold. These compounds can be classified into two groups based on their contribution to the sensorial properties of wine. Amongst the positive contributors are the polyfunctional thiols, imparting fruity aroma to wine when present at moderate concentrations (Dubourdieu et al. 2006). 3MH, its acetylated derivative 3MHA, and 4MMP are present in grapes in their precursor form, conjugated to cysteine or glutathione (Capone et al. 2010; Peyrot des Gachons et al. 2002; Tominaga et al. 1998). During fermentation yeast take up these precursors and cleave them to release free volatile thiols into the media (Grant-Preece et al. 2010; Swiegers et al. 2007; Winter et al. 2011). This process is affected by environmental conditions such as temperature and media composition (Masneuf-Pomarède et al. 2006; Subileau et al. 2008). Concentration of polyfunctional thiols in wine depends on the amount of precursor cleaved during fermentation and the resultant wine composition (Dubourdieu et al. 2006; Ugliano et al. 2011). In this study 3MH and 3MHA concentrations were increased with the addition of rehydration nutrients (Figure 1). Unlike 3MH and 3MHA, the concentration of 4MMP was not affected by the addition of nutrients at rehydration, while it significantly increased in fermentations where DAP was added. This result suggests that bioconversion of each thiol precursor may be driven by different regulatory mechanisms. Recently, a gene encoding a β-lyase enzyme, *IRC7*, was found to be the key determinant of 4MMP release. 3MH release, on the other hand, appears to be mediated by more than one gene (Roncoroni et al. 2011; Thibon et al. 2008), therefore it is reasonable to speculate that the treatments in our study have differentially regulated release of these thiols. Interestingly, while our transcription analyses were consistent with previous studies showing the downregulation of *IRC7 *by the nitrogen catabolite repression (NCR) pathway, we observed an increased concentration of 4MMP in response to DAP addition. We cannot rule out that *IRC7 *expression may have changed throughout the fermentation; nonetheless our results support the notion that thiol release is a complex process involving multiple enzymes.

Aside from bioconversion of precursors, thiols concentration in wine is highly affected by wine composition (Dubourdieu et al. 2006; Ugliano et al. 2011). Nutrients addition to the fermentation may have altered the final wine composition in a manner affecting thiol stability. In that case, the chemical difference between 3MH and 4MMP would account for their distinctive responses to each nutrient treatment.

A second class of sulfur compounds include those that impart unwanted odours and contribute negatively to wine quality (Swiegers and Pretorius 2007). An important compound of that group is H_2_S, which imparts a rotten egg aroma. H_2_S presence in wine is regarded as a sensory fault. Although the subject of H_2_S formation during fermentation is well studied, the factors leading to residual H_2_S in the final wine remain to be elucidated. Previous studies have pointed out a link between the kinetics of H_2_S formation during fermentation and amount of residual H_2_S in wine (Jiranek et al. 1996; Ugliano et al. 2009; Ugliano et al. 2010). In this study we found the supplementation of rehydration nutrients decreases the amount of residual H_2_S and affects H_2_S kinetics during fermentation. We can speculate that the decreased residual H_2_S in the final wine may be due to this altered H_2_S production kinetics, still, further study is needed in order to link between the two effects and to understand the factors affecting H_2_S during fermentation.

H_2_S is formed during fermentation as an intermediate in the biosynthesis of the sulfur-containing amino acids (pathway is illustrated in Figure 2c). This pathway involves reduction of sulfate; the most abundant sulfur source in grape must, into sulfide through the sulfate assimilation pathway and incorporation of sulfide into an amino acid precursor. Insufficient amounts of the amino acid precursor lead to accumulation and liberation of H_2_S into the media. As precursor availability derives from nitrogen metabolism, YAN concentration of the media is regarded as a key regulator of H_2_S formation (Jiranek et al. 1995).

When hydrogen sulfide formation was monitored during fermentation, we observed non-nitrogen mediated effect on H_2_S kinetics following rehydration nutrient supplementation (Figure 1d). This suggests that nitrogen deficiency is not the sole regulator of H_2_S production, in agreement with recent studies (Linderholm et al. 2008; Moreira et al. 2002; Ugliano et al. 2010), and that other nutrients may be involved. Subsequent transcription analyses supported this observation and demonstrated that regulation of H_2_S formation by rehydration nutrients did not involve the sulfate assimilation pathway (Figure 2c) because this pathway was down-regulated in response to rehydration nutrient supplementation. On the contrary, the same pathway was upregulated following DAP addition to the fermentation medium, in accordance with previous results in the literature (Marks et al. 2003; Mendes-Ferreira et al. 2010). Together, our results suggest that H_2_S produced under these conditions was formed via an alternative biochemical route. A potential activator of that route would be the tri-peptide glutathione, which was previously implicated as a source for H_2_S (Rauhut 2008; Vos and Gray 1979). The nutrient mixture contained a considerable component of GSH that was taken up by yeasts during rehydration (Figure 4b) and we also observed an upregulation of genes involved in GSH metabolism following rehydration with nutrients (Figure 3c). Supplementation of the rehydration medium with GSH altered H_2_S kinetics during fermentation (Figure 4c). Interestingly, other components of the commercial rehydration nutrient studied had a significant effect on yeast metabolic responses to GSH supplementation during this process. When GSH was added as a component of the rehydration nutrient mix, changes in H_2_S kinetics occurred during the early stage of fermentation but did not affect the final cumulative amount of H_2_S produced during fermentation (Figure 1c). On the other hand, rehydration in the presence of GSH alone resulted in a change in H_2_S kinetics throughout the fermentation process and led to a higher cumulative production of H_2_S. This difference may be associated with differences in the uptake of GSH from each medium, or reactivity of GSH with other substances of the rehydration nutrient mixture. Nonetheless, these experiments are first to demonstrate a clear effect of GSH supplementation at rehydration on the kinetics of H_2_S formation during fermentation. It is worth noting in that regard that previous studies indicated the concentration of ~50 mg/L glutathione in the grape juice is required to detect H_2_S formation from GSH (Rauhut 2008). In this study the concentration of glutathione that was carried over from the rehydration media to the grape juice was less than 1 μg/L, highlighting the importance of glutathione uptake during rehydration.

The mechanism of GSH contribution to H_2_S formation during the wine fermentation has not been elucidated. GSH is composed of the three amino acids: glutamate-cysteine-glycine. As such it contains both nitrogen and sulfur constituents, which may regulate the formation of H_2_S in different manners. When organic nitrogen was added to the rehydration medium as an amino acid mixture we did not observe changes in H_2_S kinetics during fermentation (Figure 4a), suggesting that organic nitrogen by itself did not contribute to or regulate H_2_S formation, when added at rehydration. This result points to the sulfur constituent of GSH, cysteine, as a contributor to H_2_S formation. Direct production of H_2_S from cysteine has been demonstrated previously for *S. cerevisiae *(Jiranek et al. 1995; Rauhut 2008; Tokuyama et al. 1973). Accordingly, the mechanism suggested here for H_2_S production from GSH requires GSH degradation to the individual constituent amino acids, followed by degradation of cysteine to H_2_S by an enzyme having a cysteine desulfuhydrase activity (EC 4.4.1.15, EC 4.4.1.1). This mechanism is in accordance with our phenotypic and transcriptomic results as it describes non-nitrogen mediated regulation on H_2_S formation, which is not via the sulfate assimilation pathwayIn conclusion, as wine quality can be greatly affected by the composition of sulfur compounds, this study demonstrates a potential approach for sulfur aroma management by optimising yeast rehydration conditions and providing nutrients at rehydration.

## Competing interests

The authors declare that they have no competing interests.

## Supplementary Material

Additional file 1 **Concentration of wine acids, acetate esters and higher alcohol following nutrient supplementation**. Concentration of acids, acetate esters and volatile alcohols followingthe two nutrient treatments, addition of rehydration nutrients to the rehydration media and addition of DAP to the fermentation media. Click here for file
