# Supplementary material for: Effects of rehydration nutrients on H2S metabolism and formation of volatile sulfur compounds by the wine yeast VL3
Source: AMB Express. 2011 Nov 2;1:36. doi: 10.1186/2191-0855-1-36 (PMC3226641; doi:10.1186/2191-0855-1-36)
Supplement: Additional file 1 — Concentration of wine acids, acetate esters and higher alcohol following nutrient supplementation. Concentration of acids, acetate esters and volatile alcohols followingthe two nutrient treatments, addition of rehydration nutrients to the rehydration media and addition of DAP to the fermentation media. [file 2191-0855-1-36-S1.DOC]

**Effects of rehydration nutrients on H_2_S metabolism and formation of volatile sulfur compounds in wine: role of glutathione**

Applied microbiology and biotechnology

Gal Winter^a,b^, Henschke, Paul A.^b^, Higgins, Vincent J.^a,c^, Ugliano, Maurizio^b,d^, Chris D. Curtin^b^*

^a^School of Biomedical and Health Sciences, College of Health and Science, University of Western Sydney, NSW, Australia.

^b^The Australian Wine Research Institute, P.O. Box 197, Glen Osmond, Adelaide, SA 5064, Australia.

^c^Ramaciotti Centre for Gene Function Analysis, School of Biotechnology and Biomolecular Sciences, University of New South Wales, NSW, Australia

^d^Nomacorc SA, 2260 route du Grès, 84100 Orange, France.

* To whom correspondence should be addressed

E-mail: [chris.curtin@awri.com.au](mailto:chris.curtin@awri.com.au)

**Supplementary Material – concentration of wine acids, acetate esters and higher alcohol following nutrient suppllementation**

| **Nutrient Treatment** | **Control** | | | **Rehydration Nutrients** | | | **DAP** | | |
| --- | --- | --- | --- | --- | --- | --- | --- | --- | --- |
|  | Average (µg/L) | Std (µg/L) | CV (%) | Average (µg/L) | std(µg/L) | CV (%) | Average (µg/L) | Std (µg/L) | CV (%) |
| 3-Methylbutanoic Acid | 527.08 | 94.77 | 17.98 | 227.13 | 20.18 | 8.88 | 534.68 | 79.64 | 14.90 |
| Hexanoic acid | 4998.05 | 846.58 | 16.94 | 7480.92 | 274.99 | 3.68 | 4441.72 | 134.04 | 3.02 |
| ethyl acetate | 25878.52 | 2690.57 | 10.40 | 35231.78 | 835.79 | 2.37 | 27996.32 | 1398.97 | 5.00 |
| 3-methyl butyl acetate | 1609.82 | 144.36 | 8.97 | 2410.88 | 155.56 | 6.45 | 1367.68 | 27.87 | 2.04 |
| Phenylethyl acetate | 334.42 | 40.55 | 12.13 | 149.53 | 19.45 | 13.01 | 245.35 | 15.54 | 6.33 |
| 3-methylbutanol | 175947.42 | 7506.97 | 4.27 | 101683.10 | 9436.16 | 9.28 | 181828.32 | 5663.31 | 3.11 |
